# Association of neutropenia at disease onset with severe surgical necrotizing enterocolitis and higher mortality: A retrospective study

**DOI:** 10.3389/fsurg.2022.971898

**Published:** 2022-10-11

**Authors:** Fanyue Qin, Mengjie Yuan, Chen Zhang, Chu Zhu, Huifang Dong, Falin Xu

**Affiliations:** ^1^ Department of Neonatology, The Third Affiliated Hospital of Zhengzhou University, Zhengzhou, China; ^2^ Advanced Medical Research Center of Zhengzhou University, Zhengzhou, China

**Keywords:** necrotizing enterocolitis, neutrophil, neonatal surgery, thrombocytopenia, correlation, prediction

## Abstract

**Background:**

Neutrophils are among the earliest immune cells recruited to the site of an intestinal injury, but their predictive role in the progression of necrotizing enterocolitis (NEC) has not been fully elucidated. This study aimed to evaluate if a reduction in neutrophils at the onset of NEC is associated with severe surgical NEC and/or NEC-associated deaths.

**Methods:**

This is a retrospective cohort study in which neonates underwent surgery due to NEC during 2015–2020. The data on absolute neutrophil count (ANC), before and at the onset of NEC, were collected from the complete blood count results. The primary exposure was the difference in absolute neutrophil count (ΔANC) at NEC onset. The primary outcome was severe surgical NEC, defined as the residual small bowel length after intestinal resection of <30 cm.

**Results:**

A total of 157 neonates were included in this study, of which 53 were diagnosed with severe surgical NEC. A decrease in ANC at the onset of NEC was associated with an increased probability of severe surgical NEC (crude odds ratio [OR] 1.248, 95% CI 1.107–1.407; *P* = 0.000). ΔANC (area under the curve [AUC] 0.729, 95% CI 0.653–0.797; *P* < 0.001] was a good predictor for severe surgical NEC. The addition of platelets to ΔANC at NEC onset (AUC 0.738, 95% CI 0.662–0.808; *P* < 0.001) resulted in a higher AUC and specificity for severe surgical NEC prediction than ΔANC alone. A reduction in the neutrophil count at NEC onset (ΔANC > 0) was associated with adverse outcomes (hazard ratio [HR] 3.48, 95% CI 1.64–7.36) and a lower survival probability (χ^2^ 10.63; *P* < 0.001).

**Conclusion:**

A reduction in the ANC at the onset of NEC was associated with severe surgical NEC and higher mortality. The addition of platelets to ΔANC at NEC onset resulted in a higher predictive value of severe surgical NEC. This study may provide a new insight into the bedside evaluation of NEC by analyzing data from the day of NEC onset.

## Introduction

Recent years have seen an improvement in neonatal intensive care, following which the survival rate of infants diagnosed with necrotizing enterocolitis (NEC) after small bowel resection has considerably improved using a multidisciplinary approach (1, 2). However, infants having a short residual length of the small bowel still need long-term parenteral nutrition and have poor long-term growth outcomes (3). The choice of optimal operative time is often confusing for neonatologists and surgeons due to the lack of typical radiological findings and abdominal signs, thus making it difficult to identify and diagnose progressive NEC (4).

Neutrophils are the earliest immune cells to mobilize in gastrointestinal immunity. Hence, the reduction in the neutrophil count is now considered a complex and important determinant in understanding the prognosis of severe intestinal inflammatory diseases (5, 6). Therefore, in this study, we investigated the association and predictive value of neutrophils, which is a universally available routine blood parameter with severe surgical NEC. We aimed to provide a reference for the early diagnosis and intervention of severe surgical NEC.

## Materials and methods

### Study design

This was a retrospective case-control study. The experimental design was devised and data collection and reporting of results were performed in accordance with the checklist of Strengthening the Reporting of Observational Studies in Epidemiology (STROBE) (7).

### Study setting and participants

This study included neonates who underwent surgery due to NEC in the neonatal intensive care unit (NICU) and pediatric surgery department of the Third Affiliated Hospital of Zhengzhou University between January 2015 and May 2020. The clinical, radiologic, and laboratory data of the case included in the study were obtained from medical records.

The inclusion criteria were the presence of typical NEC features, such as abdominal distention, bloody stool, intestinal pneumatosis, portal vein gas, and the intestinal pathology report. Neonates diagnosed with an additional medical condition, such as major abdominal malformation, inherited metabolic diseases, or treatment abandonment, were excluded from the study. The data were retrospectively and independently collected by two different researchers.

### Outcomes

The primary outcome was severe surgical NEC, defined as the length of the residual small bowel after intestinal resection of <30 cm. This definition refers to the study of postoperative management of NEC by surgeons and neonatologists conducted by the American pediatric surgical association (APSA) in 2018 (8). This definition was subsequently used to understand the correlation between severe surgical NEC and development of the nervous system (9). A subset of infants diagnosed with “NEC totalis” (T-NEC) was signified by a detailed review of all operative reports.

### Exposures

The primary exposure was the difference in the absolute neutrophil count (ΔANC) at the onset of NEC. ΔANC was defined as the difference in the absolute neutrophil count (ANC) obtained from the complete blood count (CBC) examination reports before and at NEC onset. The onset of NEC was defined as the time when the neonatologist suspected NEC, withdrew blood from patients, and recommended the initiation of fasting, gastrointestinal decompression, and antibiotic therapy. The ANC before NEC onset was defined as the closest ANC count detected (at 24–72 h) before NEC onset when there was no clinical sign of NEC and the patient was clinically stable. A complete course of antenatal steroids was defined as four doses of injection dexamethasone given 7 days prior to delivery (10). The partial course was also defined as prenatal glucocorticoids used once or thrice. Transfusion was defined as transfusion therapy given within 48 h before the onset of NEC (11). The duration of antibiotic exposure was defined as the number of antibiotic days prior to NEC onset.

### Data sources/measurements

We collected information concerning maternal factors, demographic characteristics, and feeding strategies. Full-volume feeds were defined according to the European Society for Paediatric Gastroenterology, Hepatology, and Nutrition Committee on Nutrition recommendations for fluid volume (12). NEC features, such as the age of onset, primary clinical presentation, and primary radiographic findings, were also recorded. Furthermore, the whole blood C-reactive protein (CRP), platelet counts (Plt) in CBC results, and lactate levels in blood gas analysis before NEC onset and on the day of NEC onset were also noted. The missing data are reported in the (Table 1). In addition, the survival rates of patients within 180 days after NEC were noted. This study received the approval of the Ethics Committee Review Board of the Third Affiliated Hospital of Zhengzhou University with a waiver of written informed consent (2022-085-01) (13).

**Table 1:** Imaging and examination characteristics of the two groups at NEC onset.^a^

	Non-severe surgical NEC (*n* = 104)	Severe surgical NEC (*n* = 53)	*Z*/χ^2^	*P*-value
ΔANC > 0	37 (35.6)	40 (75.5)	−4.61	0.000
Age at NEC onset (days)	11 (5–26)	15 (4–29)	−0.21	0.834
Pneumatosis	22 (21.2)	25 (47.2)	−3.36	0.001
Portal venous gas	13 (12.5)	18 (34)	−3.18	0.001
Pneumoperitoneum	35 (33.7)	8 (15.1)	−2.46	0.014
ANC, 10^9^/L				
Before NEC onset	3.83 (2.76–4.86)	4.1 (3.19–5.34)	−0.12	0.901
At NEC onset	3.97 (2.53–8.23)	2.12 (1.41–4.13)	−4.49	0.000
ΔANC	−0.54 (−3.74 to 1.25)	2.12 (0.88–2.84)	−5.95	0.000
Plt, 10^9^/L				
Before NEC onset	248 (187–345)	241 (221–297)	−0.17	0.867
At NEC onset	219 (156–279)	156 (109–223)	−3.46	0.001
ΔPlt	18 (−66 to 141)	84 (28–123)	−2.49	0.013
Lac (mmol/L)				
Before NEC onset	0.8 (0.5–1.1)	0.8 (0.5–1.2)	−0.30	0.764
Missing data	5 (4.8)	2 (3.4)		
At NEC onset	0.9 (0.6–1.2)	1 (0.6–1.2)	−0.40	0.686
ΔLac	−0.1 (−0.5 to 0.4)	−0.1 (−0.6 to 0.3)	−0.27	0.784
Missing data	5 (4.8)	2 (3.4)		
CRP (mg/L)				
Before NEC onset	0.52 (0.23–0.88)	0.5 (0.2–0.9)	−0.76	0.445
At NEC onset	19.4 (3.2–69.7)	32 (4.7–83.9)	−1.09	0.274
ΔCRP	−18.8 (−68.3, −2.3)	−31.7 (−85.6, −4)	−1.11	0.268

ANC, absolute neutrophil count; CRP, C-reactive protein; Lac, lactate; NEC, necrotizing enterocolitis; Plt, platelet count.

^a^ Data are expressed as *n* (%) or median (interquartile range); ANC Neutrophil, (ΔANC) Neutrophil difference = ANC before NEC onset – ANC at NEC onset.

### Methods

The patients and clinical figures were fitted with a non-normal distribution. Hence, categorical variables were reported using frequencies and percentages, and continuous variables were reported using median and interquartile ranges (IQR) (Table 2). The differences in variables between the two groups were evaluated by using the Mann–Whitney *U* test. The univariate and multivariate logistic regression models were made to estimate the association between severe surgical NEC and differences in ANC. The Box–Tidwell test was used to evaluate whether the conversion value between the continuous independent variable and the dependent variable logit was linear. Tolerance and variance inflation factors were used to determine that no multicollinearity existed among independent variables. A directed acyclic graph was drawn to identify potential confounders that could induce a causal association between ANC at the onset of NEC and severe surgical NEC (Supplementary Material 3). Receiver operating characteristic (ROC) curve analysis was applied to study the area under the curve (AUC) to predict severe surgical NEC. The postoperative survival rate was estimated and illustrated by the Kaplan–Meier method, and the differences were assessed by using the log-rank test. All tests were two-tailed, and the level of significance was set at 0.05. SPSS Statistics (version 26.0; IBM Corp., Armonk, NY, USA), MedCalc (version 20.03; Ostend, Belgium), and R software (version 4.2.0; R Foundation for Statistical Computing, Vienna, Austria) were used for statistical analyses.

**Table 2:** The perinatal outcomes of the two groups.^a^

	Non-severe surgical NEC (*n* = 104)	Severe surgical NEC (*n* = 53)	*Z*/χ^2^	*P*-value
Gestational age at birth (weeks)	31.5 (29.1–34.8)	31.14 (29.2–34.2)	−0.29	0.769
≥37	14 (13.5)	8 (15.1)		
<37, ≥32	33 (31.7)	13 (24.5)		
<32, ≥28	40 (38.5)	25 (47.2)		
<28	17 (16.3)	7 (13.2)		
Birth weight (g)	1,540 (1,103–2,090)	1,400 (1,020–2,010)	−0.81	0.420
≥2,500	15 (14.4)	11 (20.8)		
<2,500, ≥1,500	40 (38.5)	15 (28.3)		
<1,500, ≥1,000	36 (34.6)	15 (28.3)		
<1,000	13 (12.5)	12 (22.6)		
Male	62 (59.6)	35 (66.0)	−0.78	0.435
Twins	5 (4.8)	3 (5.6)	−0.03	0.978
PDA	11 (10.6)	9 (17)	−1.54	0.123
SGA	11 (10.6)	8 (15.1)	−0.82	0.413
Antenatal dexamethasone			−1.92	0.055
None	13 (12.5)	8 (15.1)		
Complete course^b^	32 (30.8)	12 (22.6)		
Partial course^c^	59 (56.7)	33 (62.3)		
Duration of antibiotic exposure (days)^d^			−1.50	0.135
None	41 (39.4)	58 (36.9)		
0–4	34 (32.7)	51 (32.5)		
≥5	29 (27.9)	48 (30.6)		
Transfusion^e^	3 (2.9)	5 (9.4)	−1.76	0.079
First feed day of life (h)	22 (12–36)	19 (11–44)	−0.09	0.927
Breast milk	58 (55.8)	23 (43.3)	−1.35	0.178
Full feeds achieved	47 (45.2)	21 (39.6)	−0.66	0.507
Enteral feed volume before onset (ml/kg)	109 (60–133)	83 (44–122)	−1.81	0.071

NEC, necrotizing enterocolitis; PDA, patent ductus arteriosus; SGA, small for gestational age.

^a^ Data are expressed as *n* (%) or median (interquartile range).

^b^ The complete course of antenatal steroids was defined as prenatal glucocorticoids used four times.

^c^ The partial course was defined as prenatal glucocorticoids used 1–3 times.

^d^ The duration of antibiotic exposure was defined as the number of antibiotic days prior to the onset of NEC.

^e^ Transfusion was defined as transfusion therapy within 48 h before NEC onset.

## Results

### Participants

**Figure 1:**
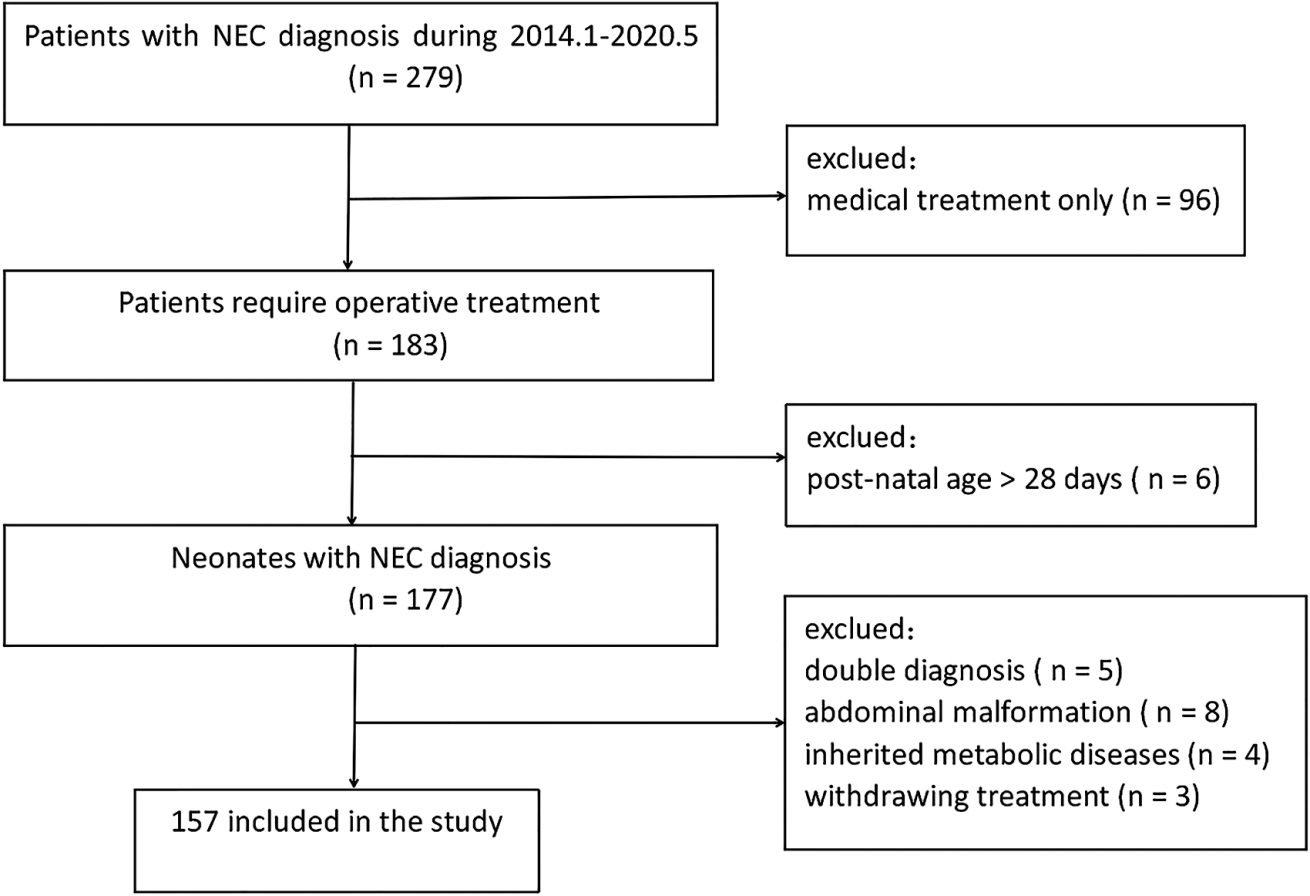
The study flow chart.

In the final analysis, 157 neonates were included. The flow chart of the study is illustrated in Figure 1. A known sepsis that subsequently developed into clinical NEC was confirmed radiologically in five patients who were excluded from the study. After a review of the medical records, it was assumed that NEC is a serious intestinal tissue injury caused by sepsis. Hence, these five patients were not included in this study.

Out of the 157 neonates with surgical NEC, 38 were transferred to the hospital for surgical treatment due to NEC, 24 were extremely preterm infants, and 22 were born full-term infants (Supplementary Material 4). The median gestational age of surgical patients was 31.3 weeks (IQR 29.1–34.7 weeks), the median birth weight was 1520 g (IQR 1100–2080 g), and the median age of NEC onset was 11 days (IQR 5–26 days). There was no noticeable difference in perinatal conditions between infants with severe surgical NEC and non-severe NEC (Table 2).

A total of 53 neonates were diagnosed with severe surgical NEC; of them, 52 underwent surgery and one infant with a gestational age of 24 + 3 weeks died immediately after a bedside abdominal puncture was performed on the infant, who suffered from severe pneumoperitoneum. In addition, out of the 53 neonates having severe surgical NEC, 8 were diagnosed with T-NEC. Infants with severe surgical NEC have a higher probability of developing pneumoperitoneum (*P* = 0.014) and a lower probability of developing pneumatosis (*P* = 0.001) and portal venous gas (*P* = 0.001) than infants with non-severe surgical NEC.

Decreased ANC was present in 77 patients at the onset of NEC; of them, 40 were severe surgical NEC. The median ANC value at NEC onset was 3.54 × 10^9^/L (IQR 1.9–6.62 × 10^9^/L), and the median ΔANC was −0.3 × 10^9^/L (IQR −2.28 to 2.11 × 10^9^/L). ANC before NEC onset did not differ between the two groups. ANC at NEC onset was lower in neonates who developed severe surgical NEC in comparison with neonates who did not (*P* = 0.000). ΔANC was higher in the severe surgical NEC group (*P* = 0.000). CRP increased in both groups at NEC onset. Plts were lower in the severe surgical NEC group and plasma lactate concentration was higher. The median Plt at NEC onset was 213 × 10^9^/L (IQR 142–266 × 10^9^/L), and the median ΔPlt was 41 × 10^9^/L (IQR −37.5 to 134 × 10^9^/L). There were significant differences in Plt and ΔPlt values at NEC onset between the two groups (*P* = 0.001, *P* = 0.013) (Table 1).

### Correlation and prediction

Upon univariate analysis, the risk factors for NEC were Plt at NEC (odds ratio [OR] 0.996, 95% confidence interval [CI] 0.993–1.000; *P* = 0.044), pneumatosis (OR 3.328, 95% CI 1.627–6.808; *P* = 0.001), portal venous gas (OR 3.600, 95% CI 1.597–8.116; *P* = 0.002), and pneumoperitoneum (OR 0.350, 95% CI 0.149–0.824; *P* = 0.016) (Supplementary Material 1).

The results of the linear test revealed a linear relationship among all continuous independent variables and the logit conversion values of the dependent variables. There was no multicollinearity for most variables. None of the interaction terms were significant among the variables studied. The analysis of ΔANC revealed that the more the ANC dropped at NEC onset, the higher the odds of developing severe surgical NEC (crude OR 1.248, 95% CI 1.107–1.407; *P* = 0.000). The possible confounding factors were selected according to the results of the univariate analysis and directed acyclic graph. The adjusted ORs are depicted in Table 3. In fully adjusted models, ΔANC remained associated with severe surgical NEC (OR 1.308, 95% CI 1.113–1.539; *P* = 0.001).

**Table 3:** Odds ratios for severe surgical NEC in patients with NEC.^a^

	OR (95% CI)	*P*-value
ΔANC at NEC onset, adjusted for gestational age, birth weight	1.268 (1.115–1.442)	0.000
ΔANC at NEC onset, adjusted for gestational age, birth weight, breast milk, PDA, SGA, age at NEC onset, first feed day of life, full feeds achieved, enteral feed volume before the onset	1.360 (1.169–1.583)	0.000
ΔANC at NEC onset, adjusted for gestational age, birth weight, breast milk, age at NEC onset, first feed day of life, full feeds achieved, enteral feed volume before onset, PDA, SGA, pneumatosis at NEC onset, portal venous gas at NEC onset, pneumoperitoneum at NEC onset, CRP at NEC onset, Plt at NEC onset	1.308 (1.113–1.539)	0.001

^a^ (ΔANC) Neutrophil difference = ANC before NEC onset – ANC at NEC onset; CRP, C-reactive protein; Lac, lactate; NEC, necrotizing enterocolitis; PDA, patent ductus arteriosus; Plt, platelet; SGA, small for gestational age.

According to the ROC analysis, the ANC, ΔANC, and Plt could predict severe surgical NEC (*P* < 0.001). ΔANC was still the most sensitive factor in predicting severe surgical NEC, with a sensitivity of 71.7%. The best cutoff value of ΔANC in predicting severe surgical NEC was >0.55  ×  10^9^/L; ANC was > .53 × 10^9^/L and Plt was >172 × 10^9^/L. Furthermore, the combination of ΔANC and Plt at onset resulted in a sensitivity of 58.5% and specificity of 81.7% for predicting severe surgical NEC (*P* < 0.001) (Table 4; Supplementary Material 2).

**Table 4:** C-statistics for the prediction of severe surgical NEC.^a^

	Associated criterion	AUC (95% CI)	SE (%)	SP (%)	PPV (%)	NPV (%)	*P*-value
ANC at NEC onset	2.53	0.720 (0.642–0.788)	58.5	75.0	65.5	78.0	<0.001
ΔANC at NEC onset	0.55	0.729 (0.653–0.797)	71.7	56.7	83.3	66.2	<0.001
Plt at NEC onset	172	0.604 (0.522–0.681)	49.1	74.0	49.1	73.6	0.032
ANC + Plt at NEC onset	0.37	0.737 (0.661–0.804)	69.8	76.3	52.1	81.4	<0.001
ΔANC + Plt at NEC onset	0.46	0.738 (0.662–0.808)	58.5	81.7	61.7	78.2	<0.001

ANC, absolute neutrophil count; AUC, area under the curve; CI, confidence interval; NEC, necrotizing enterocolitis; NPV, negative predictive value; Plt, platelet count, PPV, positive predictive value; SE, sensitivity; SP, specificity.

^a^ ANC Neutrophil, (ΔANC) Neutrophil difference = ANC before NEC onset– ANC at NEC onset.

### Clinical outcome

In 28 patients, NEC-associated death was seen within the first 180 days of life. In the severe NEC group that underwent severe surgery, 22 children died after surgery: 13 children died within 10 days of surgery because of systemic inflammatory response syndrome, shock, or disseminated intravascular coagulation; 5 died due to recurrence at 37, 63, 74, 97, and 125 days after the onset of NEC, 4 of whom underwent a second surgery; 3 patients died due to acute sepsis at 10, 16, and 19 days after surgery; and one patient died after undergoing peritoneocentesis. In the group without severe surgical NEC, only six patients died after surgery: five patients died within 10 days of surgery and only one patient died due to recurrence in the first 160 days after NEC. In addition, for T-NEC, seven patients died within a week of the surgery and only one patient survived.

**Figure 2:**
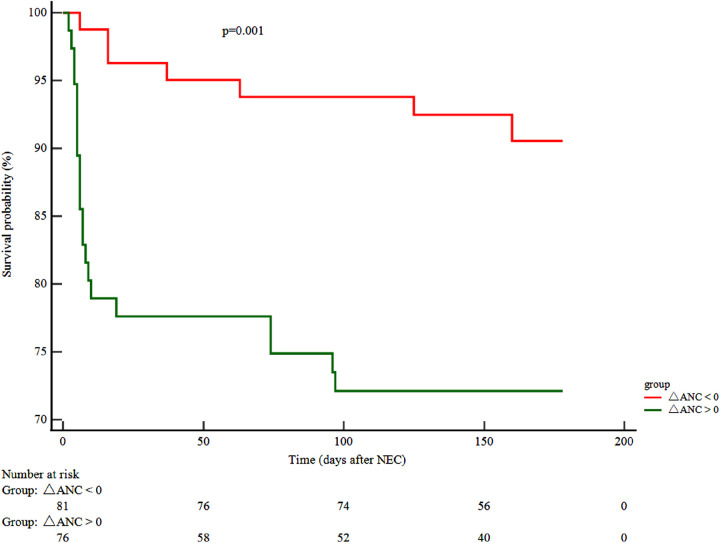
ΔANC and clinical outcome. Kaplan–Meier curve displaying the survival probability in relation to ΔANC. (ΔANC) Neutrophil difference = ANC before the onset of NEC – ANC at NEC onset. ANC, absolute neutrophil count; NEC, necrotizing enterocolitis.

Follow-up data were available for only 148 patients as 9 (5.7%) patients were lost to follow-up. A reduction in the neutrophil counts, noted at NEC onset (ΔANC > 0), was associated with adverse outcomes (HR 3.48, 95% CI 1.64–7.36) and a lower survival probability (χ^2^ 10.63; *P* < 0.001) (Figure 2).

## Discussion

### Key results

The key findings of the present study were the following: (a) a sudden reduction in the whole blood neutrophil counts at the onset of NEC was positively connected with severe surgical NEC or death—for each 10^9^/L ΔANC reduction at NEC onset, the odds for severe NEC increased by almost 25%; (b) ΔANC revealed a superior predictive validity compared with neutrophil counts at NEC ; and (c) the inclusion of Plt at the onset of NEC to ΔANC resulted in a higher predictive value for severe surgical NEC compared with ΔANC alone.

### Limitations

First, considering the retrospective trial and the small number of patients, data should be reported carefully to ensure the reliability of the statistical conclusions. Second, in current investigations, a scarcity in terms of quantifying the clinical outcomes of severe surgical NEC and T-NEC is noted, depending on subjective terms (9). The diagnosis of severe surgical NEC, particularly “NEC totalis”, continues to be difficult due to the absence of a global consensus. It is accepted that Bell-III NEC or concomitant gastrointestinal perforation is extremely severe. Nevertheless, in clinical practice, it is noted that most infants who undergo minor necrotic bowel resection mostly have a positive prognosis after they are provided standardized postoperative parenteral nutrition and home care. Thus, the content of the APSA questionnaire to highlight the “severity” was referred by us. A multicentered investigation should be conducted to optimize the definition of severe surgical NEC by quantifying mortality and long-term prognosis in infants with NEC. It would be an optimal aid for neonatologists and surgeons in decision-making. Third, data, especially on cases before the onset of NEC, are usually unavailable. In this study, multiple imputations were used to fill in the missing values. As neutrophils are influenced by gestational age and postnatal age (14), the primary focus of this study was to assess the changes in neutrophil count and not just the neutrophil count at NEC onset.

### Interpretation

NEC is clinically characterized by aggressive intestinal inflammation, suggesting that inflammatory cells, including neutrophils, may play a key role in the pathogenesis of the disease. The modified Bell criteria revealed that systemic manifestations of neutropenia were seen at the advanced stage of NEC (15). Grag et al. have previously demonstrated that children with fulminant NEC are more prone to neutropenia and thrombocytopenia (16). Julia et al. have demonstrated that surgical NEC ≥33 weeks had a percent drop in neutrophils at diagnosis of NEC (17). Experimental studies have also suggested that neutrophils are equally important in NEC as in acute intestinal inflammation. Klinke et al. have suggested that both neutrophil levels and neutrophil activation are critical components in establishing a more physiologically accurate neonatal intestinal necrosis mouse model. As compared to the hypoxia-hypothermia-formula feeding NEC model, the NEC model by changing neutrophil concentrations is more in line with the pathophysiology observed in neonates with NEC (18).

Even though the neutrophil identification from CBC is almost universally available, it is rarely used to assess prognosis on the day of making a diagnosis of NEC. This is largely due to the presence of too many potential confounders. Furthermore, neutrophil levels are more widely expressed in preterm neonates than in term neonates. This undoubtedly further complicates the assessment of the role of neutrophils in NEC (19). Detailed NEC datasets that include the confounders of gestational age and day of onset of NEC should be reported rigorously (20).

In our study, we reported information on the full-term neonates with surgical NEC and compared its characteristics with those published in the literature (Supplementary Material 4). Prospective studies have shown that the mean gestational age of preterm infants undergoing surgery is 31–37 weeks. The median gestational age of the participants in our study was 31 weeks, which is consistent with that in previous studies (21). Our study showed that full-term infants accounted for 10.3% of all NEC cases, which is similar to the previously reported data (22). Similarly, in this study, it was found that the gestational age of 28–32 weeks appeared to be a contributing factor resulting in severe surgical NEC, although no significant difference was noticed (Supplementary Material 1). This could be associated with the fact that late preterm and full-term infants generally have NEC complicated with sepsis, thus implying that these infants are severely ill from the beginning (23). Nevertheless, in multivariate analysis, it was observed that neutropenia at onset was still associated with severe surgical NEC after adding confounding factors such as gestational age. Neutropenia appears to be an independent risk factor for severe surgical NEC (Table 3).

Early identification of infants who are at the highest risk of disease progression toward surgical NEC has been a persistent research priority (24). Despite intense research over the past few decades to identify predictors of bowel necrosis, the imaging, clinical signs, and laboratory parameters still have limitations in defining the benefits of surgical treatment in children (25, 26). In this study, when the typical imaging evidence was noted, it was already too late because either the length of the viable intestine was too short or surgery could not be tolerated by the infants with severe physiologic derangement. In addition, blood biomarker studies in neonates are hampered due to the requirement of a large volume of blood and the inability to achieve a clean venipuncture due to technical difficulties. In addition, frequent monitoring of metabolic parameters in neonates with NEC is possible only on small amounts of blood obtained from heel punctures, such as CBC examination, CRP, and blood gas analysis. Hence, the implementation of clinically available routine markers for the prediction of NEC is crucial.

At present, the biomarkers used for predicting the progression of NEC primarily include Plt, CRP, white blood cell count, and lactate level (27, 28). The value of biomarkers, such as thrombocytopenia, in predicting the extent of the disease and the need for surgery is controversial. Clinically, the increment in CRP and lactic acid or the reduction in platelets is just a marker of the severe inflammatory process. Ververidis et al. revealed that severe thrombocytopenia has a sensitivity of only 69% in predicting intestinal gangrene (29). In addition, in our study, the predictive sensitivity of platelets at the onset of NEC was only 49%. Srinivasjois et al. determined that serial changes in CRP and plasma lactate levels could predict the progression of definite NEC toward surgery or death in preterm neonates (30). However, the predictive effect was present only for 48 h or more after the onset of NEC. Hence, we suggest that neutrophil values may be more sensitive than other hematologic parameters in predicting progressive NEC.

As part of the normal gut inflammatory response, neutrophils are recruited to sites of infection or inflammatory stimuli within minutes, and the response peaks at 24–48 h (31). Moreover, Ginzel et al. found that neutrophils infiltrated intestinal tissue first, and the number of neutrophils in the lamina propria was noticeably enhanced in the NEC model (32). However, in the course of severe NEC, numerous neutrophils in the peripheral blood were rapidly recruited to the intestine and peritoneum or attached to the wall of small blood vessels, resulting in a large reduction of neutrophils in the peripheral blood circulation. These statements explained the observations made by us.

Of course, the changes in neutrophil count during severe surgical NEC did not have a high specificity in our study (Table 4). Hence, it is still crucial to comprehensively assess the progress of NEC in combination with the clinical conditions. It is easy to evaluate the ANC. When the diagnosis of NEC was made, it was noted that the more the ANC was reduced, the higher the probability of occurrence of progressive NEC. This finding was extremely helpful in the clinical management of infants with NEC, especially while deciding whether the infant should undergo surgery or be transferred. Although pneumoperitoneum is the absolute indication of NEC surgery, it was found in this study that the incidence of pneumoperitoneum in infants with severe surgical NEC was extremely low (Table 2; Supplementary Material 1), which suggested that this decision could not be made easily in infants without pneumoperitoneum. Hence, in these infants, an additional severity marker, such as a change in neutrophil count, would be helpful.

## Conclusion

In conclusion, a reduction in the neutrophil count at the onset of NEC is associated with severe surgical NEC and has predictive value. The combination of ΔANC and Plt at NEC onset resulted in the highest values of AUC and specificity. Currently, a completely satisfactory model to predict the progression of NEC and bedside death is not available. However, with this study, we are hoping to provide novel insights into the clinical decision-making of NEC by collecting and analyzing data on the day of onset of NEC.

## Data Availability

The original contributions presented in the study are included in the article/**Supplementary Material**, further inquiries can be directed to the corresponding author.
