# Supplementary material for: Association of neutropenia at disease onset with severe surgical necrotizing enterocolitis and higher mortality: A retrospective study
Source: Front Surg. 2022 Oct 11;9:971898. doi: 10.3389/fsurg.2022.971898 (PMC9592859; doi:10.3389/fsurg.2022.971898)
Supplement: Supplementary file 1 [file DataSheet1.pdf]

**Supplemental material 1 Univariate analysis for severe surgical NEC**

| | Statistics | OR (95% CI) | p-Value |
|---|---|---|---|
| ANC, $10^9$/L | | | |
| at NEC onset | 3.54(1.90-6.62) | 0.783(0.682-0.899) | 0.000 |
| ΔANC | -0.03(-2.28-2.11) | 1.248(1.107-1.407) | 0.000 |
| Gestational age at birth, weeks | | | |
| ⩾37 | 22(14.0) | 1 | |
| ＜37,⩾32 | 46(29.3) | 0.689(0.234-2.030) | 0.500 |
| ＜32,⩾28 | 65(41.4) | 1.109(0.402-2.979) | 0.861 |
| ＜28 | 24(15.3) | 0.721(0.209-2.482) | 0.604 |
| Birth weight, g | | | |
| ⩾2500 | 26(16.6) | 1 | |
| ＜2500,⩾1500 | 55(35.0) | 0.511(0.192-1.360) | 0.179 |
| ＜1500,⩾1000 | 51(32.5) | 0.568(0.212-1.520) | 0.260 |
| ＜1000 | 25(15.9) | 1.259(0.417-3.800) | 0.683 |
| Antenatal dexamethasone (n%) | | | |
| None | 21(13.4) | 1 | |
| Complete course | 44(28.0) | 0.609(0.202-1.836) | 0.379 |
| Partial course | 92(58.6) | 0.909(0.342-2.418) | 0.850 |
| Breast milk (n%) | 80(51.0) | 0.632(0.325-1.231) | 0.177 |
| Transfusion (n%) | 8(5.1) | 3.507(0.805-15.283) | 0.095 |
| SGA (n%) | 19(12.1) | 1.503(0.565-3.996) | 0.414 |
| Enteral feed volume before onset, ml/kg | 100(53.7-130) | 0.994(0.987-1.001) | 0.085 |
| PDA (n%) | 20(12.7) | 2.159(0.802-5.814) | 0.128 |
| Pneumatosis (n%) | 47(29.9) | 3.328(1.627-6.808) | 0.001 |
| Portal venous gas (n%) | 31(19.7) | 3.600(1.597-8.116) | 0.002 |
| Pneumoperitoneum (n%) | 43(27.4) | 0.350(0.149-0.824) | 0.016 |
| Plt, 109/L | | | |
| at NEC onset | 213(142-266) | 0.996(0.993-1.000) | 0.044 |
| ΔPlt | 41(-38-134) | 1.001(0.998-1.004) | 0.472 |
| CRP, mg/L | | | |
| at NEC onset | 21.4(3.7-74.8) | 1.002(0.997-1.007) | 0.483 |
| ΔCRP | -19.3(-75.2, -2.5) | 0.998(0.993-1.003) | 0.477 |

*SGA small for gestational age, PDA patent ductus arteriosus.*

*The complete course of antenatal steroid was defined as prenatal glucocorticoids were used 4 times. The partial course was defined as prenatal glucocorticoids were used 1-3 times.*

*Transfusion was defined as transfusion therapy within 48 hours before onset of NEC.*

*ANC Neutrophil, (ΔANC) Neutrophil diifference = ANC before NEC-onset -ANC at NEC-onset.*

*Plt platelet, Lac lactate, CRP C-reactive protein*
