# Supplementary material for: Association of neutropenia at disease onset with severe surgical necrotizing enterocolitis and higher mortality: A retrospective study
Source: Front Surg. 2022 Oct 11;9:971898. doi: 10.3389/fsurg.2022.971898 (PMC9592859; doi:10.3389/fsurg.2022.971898)
Supplement: Supplementary file 2 [file DataSheet2.pdf]

Supplemental material 2 ROC analysis for the prediction of severe surgical NEC

A

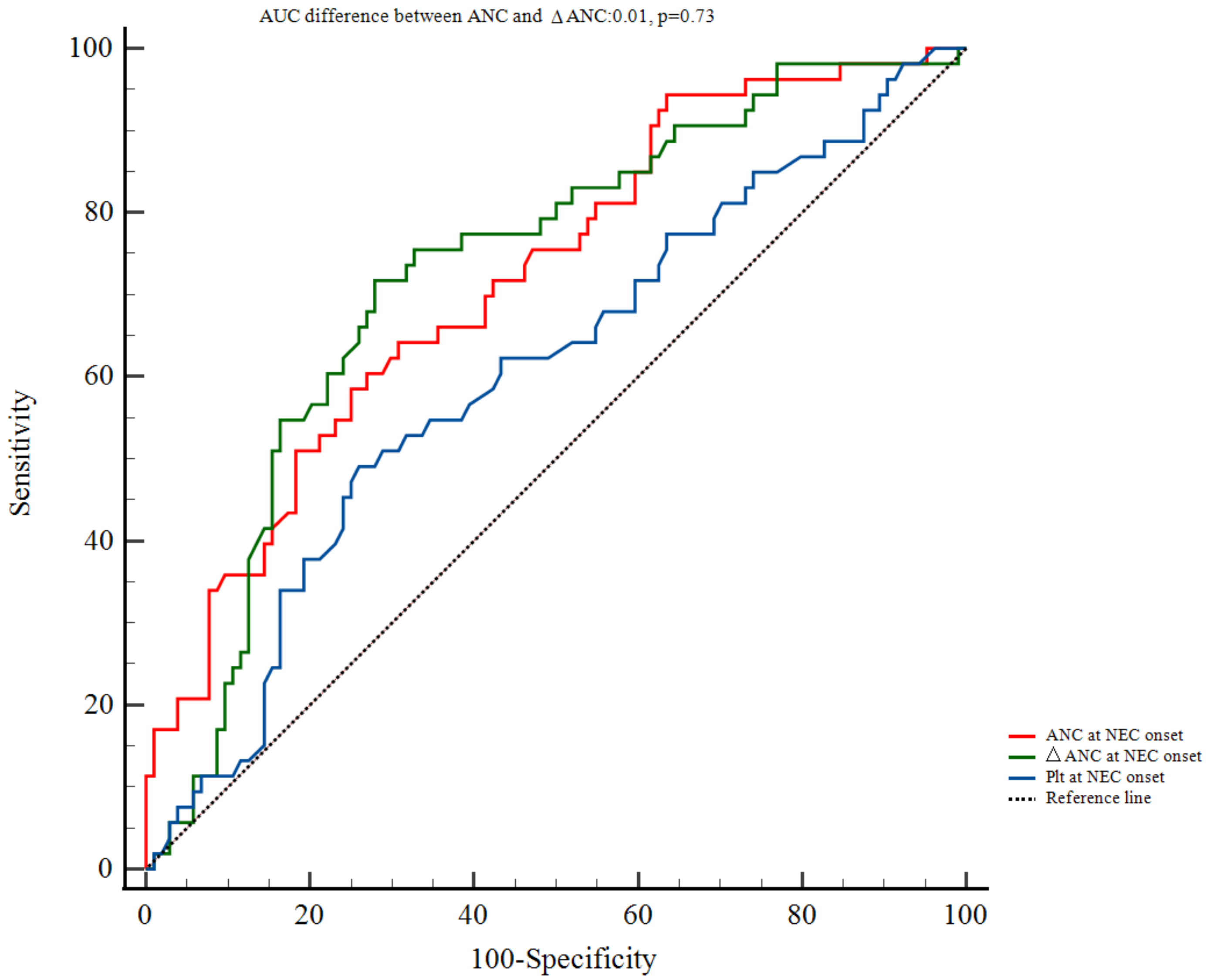

△ANC was a better predictor of severe surgical NEC (AUC 0.73, 95% CI 0.65– 0.8, $p < 0.001$)
AUC = area under the curve, ANC Neutrophil, Plt platelet
(ΔANC) Neutrophil diifference = ANC before NEC-onset – ANC at NEC-onset

B

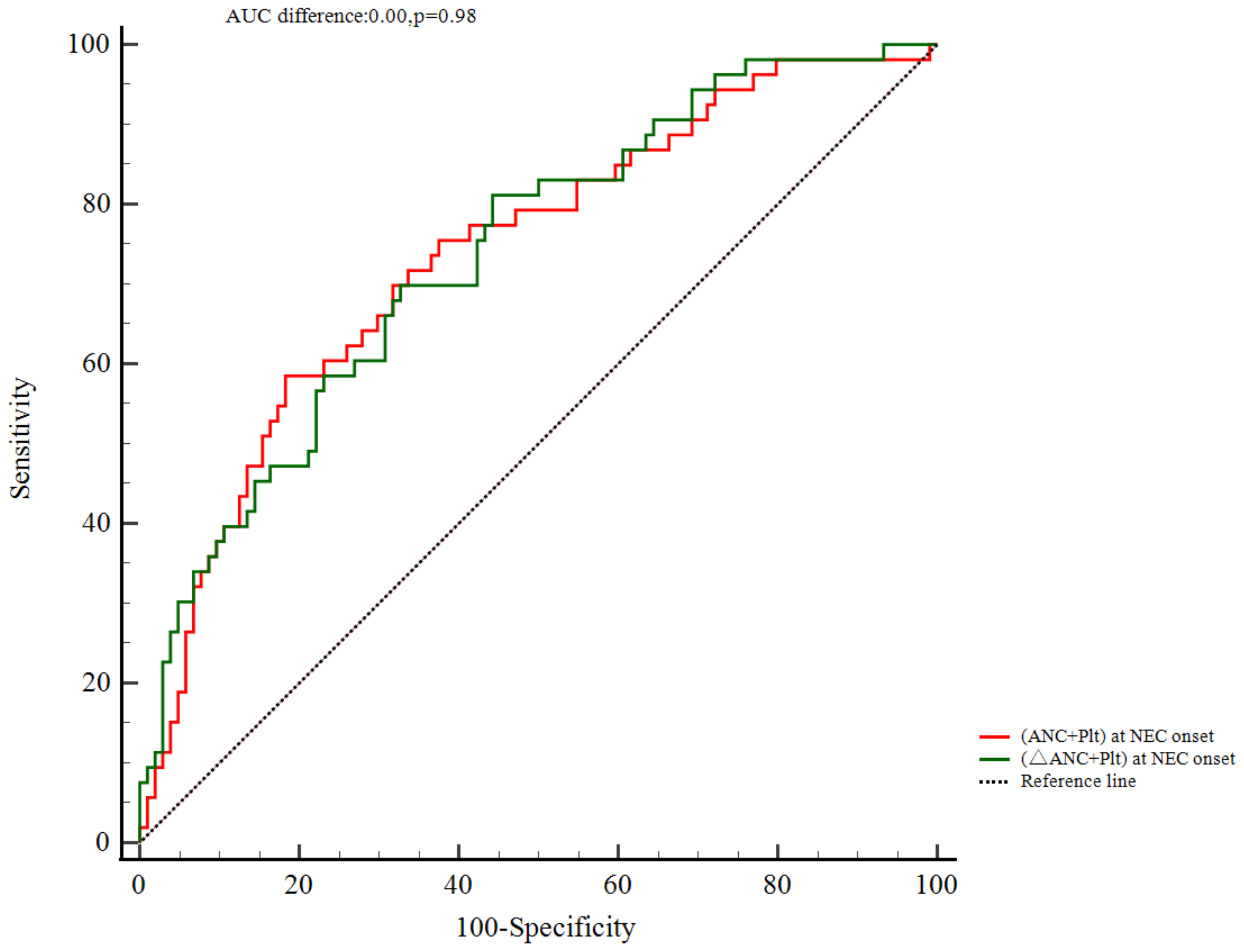

The combination of Δ ANC and Plt revealed a higher AUC(AUC 0.75, 95% CI 0.67-0.83, $p < 0.001$)
AUC = area under the curve, ANC Neutrophil, Plt platelet
(Δ ANC) Neutrophil diifference = ANC before NEC-onset – ANC at NEC-onset
