# Supplementary material for: Association of neutropenia at disease onset with severe surgical necrotizing enterocolitis and higher mortality: A retrospective study
Source: Front Surg. 2022 Oct 11;9:971898. doi: 10.3389/fsurg.2022.971898 (PMC9592859; doi:10.3389/fsurg.2022.971898)
Supplement: Supplementary file 3 [file DataSheet3.pdf]

Supplemental material 3

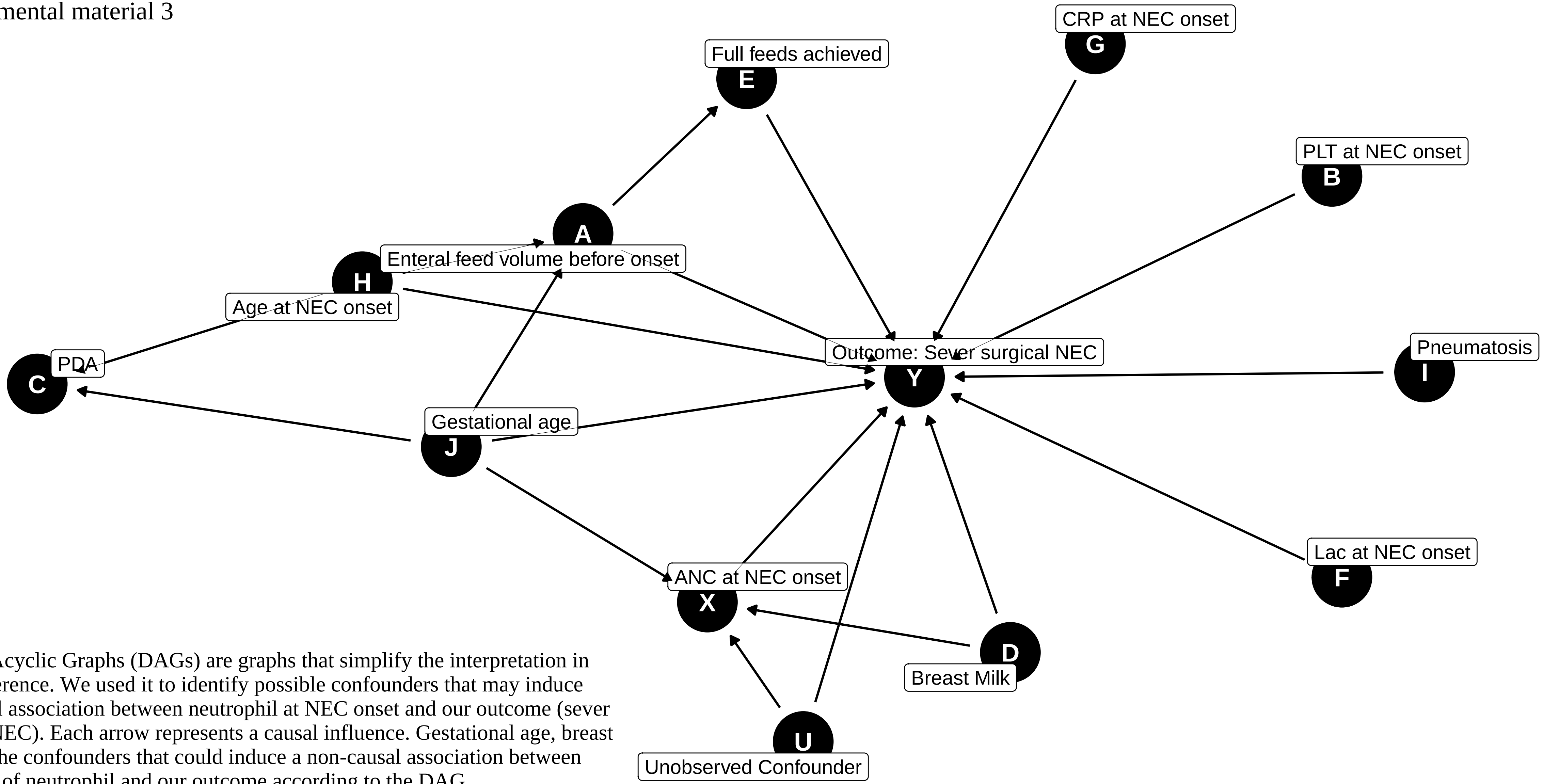

Directed Acyclic Graphs (DAGs) are graphs that simplify the interpretation in causal inference. We used it to identify possible confounders that may induce non-causal association between neutrophil at NEC onset and our outcome (sever surgical NEC). Each arrow represents a causal influence. Gestational age, breast milk are the confounders that could induce a non-causal association between difference of neutrophil and our outcome according to the DAG.
