# Supplementary material for: Association of neutropenia at disease onset with severe surgical necrotizing enterocolitis and higher mortality: A retrospective study
Source: Front Surg. 2022 Oct 11;9:971898. doi: 10.3389/fsurg.2022.971898 (PMC9592859; doi:10.3389/fsurg.2022.971898)
Supplement: Supplementary file 4 [file DataSheet4.pdf]

Supplemental material 4 Univariate analysis for severe surgical NEC in full-term infants

	Statistics	OR (95% CI)	p-Value
ANC, 10^9/L			
at NEC onset	3.73(1.44-8.53)	0.246(0.069-0.869)	0.029
ΔANC	-0.06(-5.29-2.33)	2.246(1.038-4.861)	0.040
Birth weight, g	3220(3063-3565)	1.000(0.999-1.002)	0.489
Duration of antibiotic exposure (n%)			
None	13(59.1)	-	-
0-4d	5(22.7)	5.00(0.551-45.391)	0.153
⩾5d	4(18.2)	3.33(0.319-34.830)	0.315
Breast milk (n%)	10(45.5)	0.600(0.102-3.536)	0.572
Enteral feed volume before onset, ml/kg	90(47.5-121)	0.977(0.954-1.001)	0.062
Full feeds achieved, n (%)	14(63.6)		
NEC age onset, d	4(2.8-6.5)	0.935(0.718-1.218)	0.620
Pneumatosis (n%)	5(22.7)	1.000	0.999
Portal venous gas (n%)	2(9.1)	1.000	0.999
Pneumoperitoneum (n%)	4(18.2)	1.000	0.999
Plt, 10^9/L			
at NEC onset	111(64-148)	0.994(0.984-1.004)	0.247
ΔPlt	91.5(-26.5-91.5)	1.003(0.996-1.011)	0.391
CRP, mg/L			
at NEC onset	64.8(18.4-100.4)	1.003(0.989-1.018)	0.633
ΔCRP	-57.8(-104.3, -15.9)	0.996(0.982-1.010)	0.592
Lac, mmol/L			
at NEC onset	1.0(0.8-1.3)	0.622(0.074-5.269)	0.663
ΔLac	-0.4(-0.7-0.3)	0.894(0.193-4.138)	0.886

SGA small for gestational age, PDA patent ductus arteriosus.

The complete course of antenatal steroid was defined as prenatal glucocorticoids were used 4 times. The partial course was defined as prenatal glucocorticoids were used 1-3 times.

Transfusion was defined as transfusion therapy within 48 hours before onset of NEC.

The duration of antibiotic exposure was defined as the number of antibiotic days prior to NEC onset.

ANC Neutrophil, (ΔANC) Neutrophil diifference = ANC before NEC-onset -ANC at NEC-onset.

Plt platelet, Lac lactate, CRP C-reactive protein

Participants and Clinical outcome

During the study period, a total of 253 neonates with NEC were admitted to our hospital. Ninety-six neonates of those with medical treatment were not included in our study, including four full-term neonates (Fig.1). In this study, the incidence of NEC in full-term infants was 10.3% (26/253). The median birth weight of surgical full-term

infants was 3220 (IQR 3063-3565) g. The median age of NEC onset was 4 (IQR 2.8 -6.5) days.

Of the 22 full-term infants with NEC who underwent surgery, 8 were diagnosed with severe surgical NEC. One patient was diagnosed with T-NEC and died 2 days after surgery of systemic inflammatory response syndrome.

On univariate analysis, the risk factors for NEC were ANC at NEC onset [odds ratio (OR): 0.246 (95% confidence interval (CI): 0.069-0.869), *P*=.029] and ΔANC at NEC onset [OR: 2.246 (IQR 1.038-4.861), *P*=.040].
